# An update on the biologics for the treatment of antiphospholipid syndrome

**DOI:** 10.3389/fimmu.2023.1145145

**Published:** 2023-05-19

**Authors:** Zelin Yun, Lizhi Duan, Xiangjun Liu, Qingmeng Cai, Chun Li

**Affiliations:** ^1^ Department of Rheumatology and Immunology, Peking University People’s Hospital, Beijing, China; ^2^ Beijing Key Laboratory for Rheumatism and Immune Diagnosis (BZ0135), Peking University People’s Hospital, Beijing, China; ^3^ Department of Rheumatology and Immunology, Gangkou Hospital of Hebei Port Group Company Limited, Qinhuangdao, Hebei, China

**Keywords:** biologics, antiphospholipid syndrome, eculizumab, rituximab, belimumab, daratumumab, anti-TNF-α antibodies, obinutuzumab

## Abstract

Antiphospholipid syndrome (APS) is a systemic autoimmune disease characterized by thrombosis and pregnancy morbidity with the persistent presence of antiphospholipid antibodies (aPLs). Although anticoagulation is the primary treatment for APS, it fails in approximately 20-30% of obstetric APS cases and more than 30% of thrombotic APS cases. Therefore, there is a need for new, targeted treatments beyond anticoagulants. Biologics, such as rituximab and eculizumab, have been recommended for refractory catastrophic APS. This review focuses on the recent advancements in the pathogenesis of APS and explores the potential of targeted treatments, including eculizumab, rituximab, belimumab, daratumumab, obinutuzumab, and anti-TNF-α antibodies, for APS management.

## Introduction

Antiphospholipid syndrome (APS) is a systemic autoimmune disease, characterized by thrombosis or recurrent pregnancy morbidity, with the persistent presence of antiphospholipid antibodies (aPLs) (1, 2). APS can occur as an isolated condition (primary APS, PAPS) or secondary to systemic lupus erythematosus (SLE) or other rheumatic diseases (SAPS) (3, 4).

The clinical manifestations of APS include thrombosis, obstetrical complications and “noncriteria” manifestations (2). Thrombosis can cause occlusive events in venous, arterial, or microvascular systems (5). Catastrophic APS (CAPS) affects approximately 1% of APS patients and can cause multiple thromboses of medium and small arteries, leading to fulminant multiple vital organ dysfunction (6–9). Obstetrical complications often involve unexplained, consecutive spontaneous abortions, fetal death, or premature birth due to eclampsia, severe preeclampsia, and intrauterine growth restriction (3, 10, 11). In addition, other clinical manifestations, known as “noncriteria” manifestations, include thrombocytopenia (2), hemolytic anemia (5), livedo reticularis (12), accelerated atherosclerosis and cardiac valve disease (13), nephropathy (14), neurological impairment (2), and bone necrosis (12).

Anticoagulation therapy is considered a fundamental cornerstone of APS management (15). However, conventional prevention and treatment strategies fail in approximately 20-30% of obstetric APS and more than 30% of thrombotic APS cases (16, 17). Furthermore, traditional medications are often ineffective in treating CAPS, refractory APS, and noncriteria manifestations (1, 2, 18–20).

The understanding of APS pathogenesis has grown in recent years, prompting investigation into new targeted therapies (2, 7). Multiple mechanisms have been proposed, such as B cell-mediated production of aPLs, particularly anti-β2-glycoprotein I antibody (21, 22); activation of complement (23–26); and stimulation of endothelial cells (27), platelets (28, 29), neutrophils (30, 31), and monocytes (32). This review focuses on biologics as potential targeted therapies for APS based on its underlying mechanisms. We will discuss possible biologics beyond antithrombotic agents (**Table 1**).

**Table 1:** Biologics used in the management of APS patients. .

Medication	Mechanism	Perspective
Eculizumab	Complement 5 inhibitor	For CAPS refractory to standard treatment, CAPS receiving kidney transplantation, acute TMA in patients with aPL-related nephropathy, APS during pregnancy, or pediatric CAPS (2, 33, 34)
Rituximab	Type I anti-CD20 monoclonal antibody	For thrombocytopenia, hemolytic anemia, or other aPL-mediated hematological and microthrombotic manifestations or noncriteria manifestations; an alternative option for CAPS which is refractory to standard treatment, refractory obstetric APS, and pediatric CAPS (2, 33, 35)
Obinutuzumab	Type II anti-CD20 monoclonal antibody (B-cell depletion mainly *via* DCD)	Alternative option for rituximab in APS (36)
Belimumab	BAFF/Blys inhibitor	Potential treatment for aPL-positive patients, or primary APS with high thrombotic risk (37–39)
Daratumumab	Anti-CD38 monoclonal antibody	Potential treatment for refractory APS (40, 41)
Zanubrutinib	BTK inhibitor	Unclear, evidence still being collected
Anti-TNF-α therapy	Anti-TNF-α monoclonal antibody: adalimumab, certolizumab	In refractory obstetric APS (42)

CAPS, catastrophic antiphospholipid syndrome; TMA, thrombotic microangiopathy; APS, Antiphospholipid syndrome; DCD, direct cell death; BAFF/Blys, B cell activating factor/B-lymphocyte stimulator; BTK, bruton tyrosine kinase.

## Pathogenesis of APS

**Figure 1:**
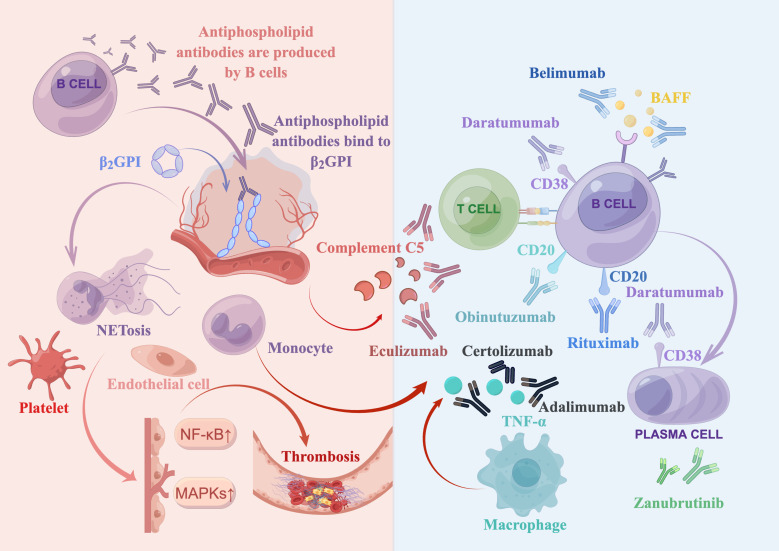
Summary of antiphospholipid syndrome pathogenesis and biologics treatments. Antiphospholipid antibodies, produced by B cells, bind to open and immunogenic β2-glycoprotein I (β2GPI) on the surface of endothelial cells. This leads to the activation of various target cells, such as complement cells, platelets, monocytes (including macrophages that secrete TNF-α), and neutrophils (which release neutrophil extracellular traps [NETosis]). Moreover, it upregulates the mitogen-activated protein kinase (MAPK) and nuclear factor kappa B (NF-κB) pathways, ultimately resulting in thrombosis. Several biologics have been developed to target various factors involved in this process. These include a complement 5 inhibitor (eculizumab), a type I anti-CD20 monoclonal antibody (rituximab), a type II anti-CD20 monoclonal antibody (obinutuzumab), a B cell activating factor (BAFF) inhibitor (belimumab), an anti-CD38 monoclonal antibody (daratumumab), anti-TNF-α monoclonal antibodies (adalimumab and certolizumab), and a bruton tyrosine kinase (BTK) inhibitor (zanubrutinib, which is currently being investigated in clinical trials).

The pathogenesis of APS supports the use of biologics as a targeted treatment approach. A brief overview is presented in **Figure 1**. In APS, aPLs primarily target β2-glycoprotein I (β2GPI), a plasma protein that binds to phospholipids. Binding of aPLs to β2GPI on the surface of endothelial cells upregulates the expression of prothrombotic cellular adhesion molecules, such as E-selectin and tissue factor (TF) (43). Notably, aPLs against β2GPI disrupt the binding of annexin A5 to phospholipid bilayers, which accelerates coagulation reactions. Annexin A5 is an anticoagulant that binds to phospholipid bilayers, impeding coagulation reactions. It does so by forming an anticoagulant shield that hinders the accessibility of anionic phospholipids (44, 45). In addition, aPLs binding to β2GPI suppress the inhibitors of tissue factor pathway (46), reduce the activity of protein C (2), and activate complement (2).

The translocation of aPLs into late endosomes, aided by annexin A2 and multiple toll-like receptors, may contribute to various pathogenic effects (47). These include the activation of multiple target cells through mitogen-activated protein kinases (MAPKs) and the nuclear factor kappa B (NF-kB) (48, 49). Platelets also play a critical role in the prothrombotic interactions between aPLs and endothelial cells (50). Additionally, active neutrophils release tissue factor, neutrophil extracellular traps (NETosis) and interleukin-8 (IL-8) that are involved in thrombosis (2, 30, 31). Furthermore, monocytes in patients with APS can express high levels of tissue factor (2). Besides, aPLs may induce upregulation of the mechanistic target of rapamycin (mTOR) complex on endothelial cells, which is associated with vasculopathy (2).

To summarize, aPLs produced by B cells bind to anionic surfaces of cell membranes and convert the closed β2GPI to the open, immunogenic conformation. *Via* translocation or other interaction mechanism, aPLs induce a proinflammatory or prothrombotic phenotype of endothelial cells, complement, and other cells (such as platelets, neutrophils, and monocytes). This leads to inflammation, vascular thrombosis, pregnancy complications, and multiple organ dysfunction through multiple mechanisms, such as reduction of protein C activity and suppression of tissue factor inhibitor (2, 33).

## Rituximab

### Mechanisms of rituximab

Rituximab is a chimeric monoclonal antibody that specifically targets CD20 on B cells. Its use in APS is based on the crucial role of B cells in the pathogenesis of the disease (51). Studies have indicated that rituximab may lower aPL titers, associated with B-cell regulatory effects (as rituximab cannot deplete plasma cell directly) (51, 52). In addition, rituximab may inhibit the expression of inducible co-stimulator (ICOS), which can suppress the activation of T helper cells in the development of APS (53, 54).

### Application of rituximab in APS

Rituximab is recommended for use in APS (12), particularly in cases of refractory CAPS (18), or CAPS with noncriteria manifestations such as acute kidney injury and severe thrombocytopenia (55–57). A real-world study of 22 APS patients who received either standard (≥1000 mg overall) or low (<1000 mg overall) dose rituximab showed that after a 6-month follow up, 19 patients achieved varying degrees of remission, with significant decreases in anticardiolipin antibody (aCL) titer and erythrocyte sedimentation rate level (58). Additionally, a recent case report suggested that rituximab may improve renal function and result in a negative aPLs profile in patients with refractory CAPS (59).

Antiphospholipid antibody levels have been shown to be significantly reduced in refractory cases following treatment with rituximab (5). In a systematic review of 20 patients with CAPS who received rituximab in combination with a first-line treatment for aggressive clinical presentation, vascular disease, or noncriteria manifestations, fifteen of them recovered from the acute episode, and 4 patients achieved a negative aPLs profile (60). Even without anticoagulation, rituximab has been shown to improve kidney function and cerebral edema in CAPS patients (61). In a retrospective study of 63 patients with SLE-associated APS, 6 patients who received rituximab therapy showed no relapse of thrombosis and a reduction in lupus activity (62). A study of 24 PAPS patients receiving rituximab found a 75% response rate (63). Furthermore, rituximab has been shown to improve multiple vascular or noncriteria manifestations in APS, including venous thrombosis (52, 64–67), arterial thrombosis (68), thrombocytopenia (66, 69), hemolytic anemia (70), pulmonary hemorrhage (71, 72), CAPS (71, 73), and overlap syndrome (74). Additionally, rituximab may benefit APS patients with concurrent malignancy (75). Notably, in a pregnant woman with primary APS who had severe thrombocytopenia and did not respond conventional therapy, weekly rituximab administration from 12 to 15 weeks of gestation increased platelet count while decreasing aCL titer, resulting in a successful pregnancy without preeclampsia or fetal growth restriction (76). Therefore, rituximab may be able to control disease activity and reduce pregnancy complications, particularly for refractory APS cases.

### Limitations

After receiving rituximab retreatment, two patients reported a thrombotic exacerbation. In both cases, it is believed that the immune complex, composed of rituximab and human anti-chimeric antibody (HACA), activated the complement process, resulting in a prothrombotic state in APS. Additionally, it is possible that infusion reactions contributed to the development of transverse myelitis in one of the patients (77). It is important to note that placental transfer of rituximab can affect the development of fetal and neonatal B cells, which could increase susceptibility to infections. Therefore, rituximab should be discontinued at least 6 months before conception (78). Other potential complications of rituximab treatment include neurological injury, infections, recurrence of arterial thromboembolic events, and rarely severe bone pain (79). However, long-term investigations have shown that adverse effects were consistent between the placebo and rituximab cohort, and serious infections or infusion reactions did not increase over time (80).

Because rituximab does not directly eliminate plasma cells, its use may lead to insufficient suppression of aPL-producing plasma cells in patients with APS (81). In combination therapy, other treatments targeting plasma cells may be considered. Generally, rituximab is not recommended as a first-line treatment for CAPS patients due to uncertainty regarding long-term efficacy or adverse effects, as well as high cost (12, 82). The widespread utility of rituximab in aPL-positive patients still requires further investigation.

## Obinutuzumab

### Mechanisms of obinutuzumab

Obinutuzumab is a type II anti-CD20 monoclonal antibody that can induce potent direct cell death (DCD) by rupturing lysosomes (83). Due to its ability to avoid rapid internalization like rituximab, this typical type II anti-CD20 antibody may induce more effective antibody-dependent cellular cytotoxicity (ADCC) and antibody-dependent cellular phagocytosis (ADCP) (84).

### Alternative option for rituximab

Previous studies have shown that obinutuzumab was more effective than rituximab at inducing B cell depletion during *in vitro* whole blood assays, even in the presence of excess B cell activating factor (BAFF) (84, 85). This suggests that obinutuzumab may be a viable treatment option for APS cases that are resistant to rituximab. In fact, a patient with APS and SLE who was resistant to rituximab showed a remarkable positive response to obinutuzumab treatment (36).

### Limitations

Obinutuzumab has shown promise as an alternative to rituximab in patients with refractory APS; however, larger clinical trials and additional data are necessary to confirm potential adverse effects. It is important to note that the impact of obinutuzumab on pregnancy or conception is currently unknown.

## Belimumab

### Mechanisms of belimumab

Belimumab is a monoclonal antibody targeting the soluble circulating BAFF (7). As previously mentioned, B cells play a crucial role in the pathogenesis of APS. It is noteworthy that APS patients have increased levels of BAFF (86, 87). Additionally, research on murine models has shown that inhibiting BAFF can lead to B cell depletion and an improvement in clinical manifestations (51, 88).

### Reducing aPL titers and ameliorating vascular symptoms

Belimumab has recently been found to induce negativity for aPLs in SAPS patients (37). Although initially used in 2 patients with microthrombotic APS manifestations with incomplete response, both patients experienced clinical improvement and stopped taking corticosteroids (38). A *post hoc* analysis of two randomized placebo-controlled trials in SLE confirmed its effectiveness in reducing aPLs in APS (39). Belimumab may be beneficial in managing APS patients with high thrombotic risk or aPL-positive patients with microthrombotic manifestations (87). Moreover, belimumab has been shown to significantly improve thrombocytopenia in an APS patient who was unresponsive to corticosteroids and rituximab (89).

### Limitations

Limited evidence has demonstrated that belimumab is not teratogenic (90), however, current evidence is insufficient to be completely confident that it is compatible with pregnancy; therefore, the use of belimumab should be stopped at conception (78). Currently, belimumab may be considered as a management strategy for severe maternal disease in pregnancy if no other pregnancy-compatible drugs are suitable (78, 90).

## Eculizumab

### Mechanisms of eculizumab

Unrestricted activation of the complement cascade is involved in CAPS (91–93). The formation of membrane attack complex (MAC) triggers endothelial cell apoptosis, expression of inflammatory cytokines, vascular basement membrane collagen exposure, and platelet aggregation (6, 7, 94). During this process, eculizumab can interrupt formation of MAC by preventing the cleavage of complement C5 (19, 95).

### Efficacy in CAPS treatment

Eculizumab can improve outcomes of refractory CAPS described in previous case reports (18, 96–102). When rituximab and immunoglobulins fail to improve platelet count and renal function, eculizumab may be an effective alternative supplement (103, 104). It has been reported that thrombocytopenia or kidney injury in CAPS patients with thrombotic microangiopathy (TMA) may respond to eculizumab (91, 105). A recent systematic review of the efficacy of eculizumab in treatment of TMA confirmed that it resulted in 100% hematological response and 85% kidney recovery (106). In patients with CAPS, eculizumab has resulted in prevention of recurrent APS and rescuing renal allografts after kidney transplantation (96, 98, 107–111). Interestingly, eculizumab may prevent TMA associated with CAPS caused by COVID-19 infection (112). Therefore, eculizumab may be considered effective in APS patients with CAPS to prevent complications.

### Application in obstetric APS

Previous case reports have shown no obvious fetal or maternal complications when eculizumab is used during pregnancy (113–115). Recently, two APS patients were reported to have received eculizumab treatment at the end of their pregnancies, demonstrating successful prevention of potentially fatal APS-related complications (116, 117). There was no thrombosis, detectable organ damage, and infectious complications during the postpartum period, with negligible levels of eculizimab detected in the infant (116). The aforementioned case reports revealed that eculizumab may be a viable option for APS treatment during pregnancy and delivery. However, more research is required to ensure its safety and efficacy.

### Limitations

Currently, clinical data consists mainly of case reports or series, which may contain significant bias. The 16th International Congress on aPL Task Force Report on APS Treatment Trends highlighted the importance of the addition of eculizumab for the treatment of CAPS, refractory APS with TMA, and obstetric APS (42). However, it remains to be proved whether general aPL-positive patients will respond effectively to eculizumab (92).

In addition, there are safety considerations in the administration of eculizumab, including *Neisseria meningitis* infections (118) and other encapsulated organisms (e.g., *Streptococcus pneumoniae* and *Haemophilus influenza*). Therefore, patients should be vaccinated prior to eculizumab treatment (119). Another concern is the extremely high cost, which exceeds $500,000 per year (120). Due to the high economic burden, the use of eculizumab in prevention of CASP should only be available when standard treatments have failed.

## Daratumumab

### Mechanisms of daratumumab

CD38 is a glycoprotein which is highly expressed on transmembrane region of plasma cells, functioning as an adhesion molecule, ectoenzyme, and receptor for activation or proliferation signals (121, 122). CD38 antibodies can attack plasma cells directly (through disruption of calcium influx and signal transduction) as well as through Fc-dependent immune-effector mechanisms (complement-dependent cytotoxicity [CDC], ADCC, and ADCP) (122–124).

### Application in autoimmune-mediated disease

In recent years, daratumumab has been employed in management of autoimmune-mediated diseases, including APS, SLE, and rheumatoid arthritis (RA) (125). Daratumumab has been effectively used to deplete plasma cells and plasmablasts of patients with SLE and RA in a dose-dependent manner *ex vivo* (125). A clinically significant improvement was observed in a patient with APS who had recurrent venous thromboembolic events despite anticoagulant therapy. The aPL levels declined significantly and continued to decrease over the next three months (40).

### Combination therapy with rituximab

A new drug-free macromolecular therapy has been developed to simultaneously target CD20 and CD38. This therapy consists of crosslinked rituximab and daratumumab using biorecognition of the morpholino oligonucleotide-modified antibody Fab’ fragment and a multivalent effector motif. The goal of this therapy is to address the limitations of standard monoclonal antibodies and induce better ADCC and CDC effects. The complementary effects have been observed in terms of apoptosis induction and degree of synergism (126).

### Recommendations and limitations

Daratumumab may be suitable for APS patients who are unresponsive to anticoagulant therapy and standard immunosuppression (40, 41). Due to daratumumab’s ability to only suppress long-lived plasma cells, sustainable responses will rely on disallowing the regeneration of autoreactive plasma cells. Therefore, long-term maintenance therapies, such as belimumab may be required (41). While only a few case reports have provided rationales for targeting of CD38 in autoimmune diseases, large clinical trials, as well as appropriate treatment schedules and populations are still needed (41). It is worth noting that the adverse effects of daratumumab on pregnancy or conception remain unknown.

## Anti-TNF-α therapy

### Mechanisms of anti-TNF-α therapy

APLs may increase the expression of TNF-α by stimulating monocytes (127), which can lead to an increase in tissue factor production (128). *In vitro* studies have shown that adalimumab, a TNF-α blocker, completely inhibits anti-β2GPI-induced TF expression in monocytes (129). Studies conducted on mouse models have demonstrated that elevated levels of TNF-α levels in placental tissues are linked with abnormal placenta and pregnancy loss, and that blocking TNF-α can improve endothelial dysfunction and prevent pregnancy loss (130–132).

### Application in obstetric APS

In a recent case series, eighteen aPL-positive women with obstetric APS which were refractory to low molecular weight heparin, aspirin, and hydroxychloroquine were treated with adalimumab or certolizumab. Positive obstetric results were obtained in 70% of patients. TNF-α blockers were all well tolerated without adverse effects (133).

### Limitations and adverse effects

There have been reports of APS induction during anti-TNF therapy (134–136). While recent guidelines indicate that anti-TNF-α drugs can be used safely throughout pregnancy due to their very low level of transplacental transit (78), it is important to note that TNF-α blockers may have potential adverse effects (42, 137). Therefore, the rational use of these drugs should be limited to refractory cases of APS.

## Clinical trials for various monoclonal antibodies

We have compiled information on clinical trials for various biologics that are either completed or still recruiting. This is due to the high-level of evidence classification and recommendation in clinical trials. Some of these biologics have already been thoroughly introduced above, while others may be simply included due to their presently unknown effects.

A completed phase II clinical trial focused on eculizumab enabling renal transplantation in patients with a history of APS or CAPS, but no results were posted (clinicaltrials.gov#: NCT01029587). In an open-label, prospective pilot study of rituximab in 19 patients with primary APS, although the decrease of aPL titers could not be observed, the rituximab was found to be effective in controlling noncriteria manifestations such as skin ulcers, nephropathy, cognitive dysfunction, and thrombocytopenia. Twelve serious adverse events involving hospitalization in 7 patients were recorded (79). An ongoing open-label, prospective, phase II descriptive pilot trial is evaluating belimumab therapy for refractory or noncriteria manifestations of APS (clinicaltrials.gov#: NCT05020782). Additionally, a phase II clinical trial is currently evaluating the addition of certolizumab to usual treatment (a heparin agent and low-dose aspirin) in pregnant women with APS (clinicaltrials.gov#: NCT03152058).

Bruton tyrosine kinase (BTK) plays a significant role in regulating B cell proliferation, survival, differentiation, and cytokine expression (138), as well as influencing platelet activation (139). As such, zanubrutinib, a BTK inhibitor, is being studied in a prospective, single-arm, open-label clinical trial for the treatment of APS with secondary thrombocytopenia (clinicaltrials.gov#: NCT05199909). Furthermore, an open-label, phase II trial investigating the use of the complement C5 inhibitor ALXN1007 for the treatment of noncriteria manifestations of APS was terminated due to low patient enrollment (clinicaltrials.gov#: NCT02128269).

## Conclusions

Although anticoagulation remains the cornerstone of APS treatment, significant attention is being drawn towards studying a number of biologics. It is important to note that biologics are primarily reserved for patients with refractory APS or CAPS. However, the accuracy of biologics make them a promising option for the development of optimal therapies for personalized medicine. Innovative biologic-focused therapeutic approaches are being investigated to treat APS, which may ultimately reduce mortality rates among individuals with APS.

## Author contributions

ZY and LD: reviewing of literature, performing analysis, writing of the original draft and editing. QC and XL: reviewing of literature. CL: conceptualization, methodology, supervision, manuscript editing. All authors contributed to the article and approved the submitted version.
